# Investigation on Microstructure of Beetle Elytra and Energy Absorption Properties of Bio-Inspired Honeycomb Thin-Walled Structure under Axial Dynamic Crushing

**DOI:** 10.3390/nano8090667

**Published:** 2018-08-27

**Authors:** Jianxun Du, Peng Hao

**Affiliations:** 1 School of Air Traffic Management, Civil Aviation Management Institute of China, Beijing 100102, China; 2 School of Mechanical Engineering, Tianjin University, Tianjin 300072, China; cauc_haopeng@163.com; 3 School of Aeronautic Engineering, Civil Aviation University of China, Tianjin 300300, China

**Keywords:** elytra, microstructure, impact loading, aluminum alloy, hierarchy order

## Abstract

The beetle elytra requires not only to be lightweight to make a beetle fly easily, but also to protect its body and hind-wing from outside damage. The honeycomb sandwich structure in the beetle elytra make it meet the above requirements. In the present work, the microstructures of beetle elytra, including biology layers and thin-walled honeycombs, are observed by scanning electron microscope and discussed. A new bionic honeycomb structure (BHS) with a different hierarchy order of filling cellular structure is established. inspired by elytra internal structure. Then the energy absorbed ability of different bionic models with the different filling cell size are compared by using nonlinear finite element software LS-DYNA (Livermore Software Technology Corp., Livermore, CA, USA). Numerical results show that the absorbed energy of bionic honeycomb structures is increased obviously with the increase of the filling cell size. The findings indicate that the bionic honeycomb structure with second order has an obviously improvement over conventional structures filled with honeycombs and shows great potential for novel clean energy absorption equipment.

## 1. Introduction

To ensure safety during a collision, the impact energy must be orderly absorbed or completely dissipated. Many thin-walled structures with kinds of cross-sectional shapes are applied as crashworthy components because they are effective in energy absorption. In order to investigate the energy-absorbing properties of thin-walled structure, many studies has been carried out through theoretical analysis [1,2,3], numerical simulations [4,5,6], and experimental tests [7,8,9].

Thin-walled structures with different cross-sectional shapes have obviously differences on the mechanical behavior. From single tube to multiple tubes, configuration such as square tube [10,11], circular tube [12,13], rectangular tube [14,15,16], pyramidal tube [17], hexagonal tube [18,19], and conical tube [20,21] have been researched widely. The thin-walled structure with cross-sectional shapes including 12-sided and 16-sided star have also been investigated in previous works [22]. The results indicate that the mechanical behavior changed dramatically with different kinds of inward corners. Many kinds of column structures with foam-filed design have been investigated in the previous works. A square steel tube filled with honeycomb structure has been adopted for energy absorption and safety in the protective field by numerical simulation [23]. The numerical results indicate improvements on all objective functions compared to the original. In order to make the constraints for the optimization and guidelines, Bollen et al. discussed the different processing methods that have been assessed for the fabrication of the hybrids [24]. Mozafari et al. [25] presented a reinforced, light-weight sandwich structure with filling foam technique and studied how the refined one can absorb energy better. Xie et al. [26] proposed a heat capacity method to solve the problem of phase change. The parameter studies of some sandwich structures have also been performed under crushing. Baroutaji et al. [27] proposed a sandwich structure device with an aluminium foam core in the thin-walled circular tubes and developed the numerical simulation under static loading conditions. The results of different fiber orientations and different thicknesses in the skins of the sandwich have been calculated by using theoretical methods [28].

**Figure 1:**
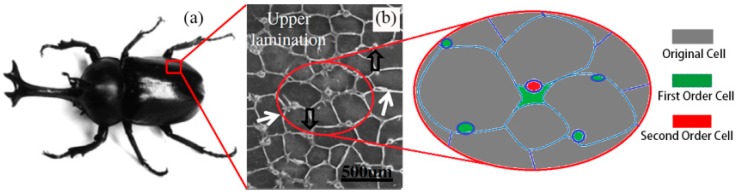
The beetle elytra and its microstructure: (**a**) the beetle *Allomyrina dichotoma*; (**b**) micromorphology of honeycomb structure with columns. “Reproduced with permission from [CARBOHYD POLYM]. Elsevier, 2013.”.

Although many works of energy absorption of thin-walled structure have been carried out, the internal structures of beetle elytra, which could resist external loading and absorb impact energy, still has not been investigated systematically and deeply. A. Zaheri et al. [29] demonstrate the adaptive behavior in helicoidal architectures by performing a mechanistic analysis of the changes occurring in the cuticle of the figeater beetle (*Cotinis mutabilis*) during its life cycle. They tested the 3D printing samples and carried out a systematic analysis of the effect of pitch angle in the inherent mechanics of helicoidal architectures and found that improved isotropy and enhanced toughness at lower pitch angles, highlighting the flexibility of the helicoidal architecture. M. Vural et al. [30] presented an experimental investigation on the compression behavior of balsa wood with different densities of specimens. The results indicated that the compressive strength of balsa wood increases with increasing density. The failure of low-density specimens is governed by elastic and/or plastic buckling, while kink-band formation and end-cap collapse dominate in higher density balsa specimens. B. Koohbor et al. [31] used ultra high-speed photography in conjunction with digital image correlation and proposed that the substantial variation across different scales is used to explain the contribution of the strength and modulus of parent polymer material in the cellular scale deformation and failure response of the specimen, as well as load bearing and strong strain rate sensitivity of the foam.” This part has been added to the introduction. Chen et al. [32] has proposed a new honeycomb column structure which was observed in beetle elytra, as shown in Figure 1. The numerical results on the mechanical behavior of bionic honeycomb structures reinforced by unequal length of fibers have been discussed. On the basis of Chen’s study, Du et al. carried out a series of investigations of the energy absorption ability of the bionic structure inspired by ladybird beetle elytra under low velocity impact [33,34,35,36]. In present work, two types honeycombs with different filling hierarchy order were proposed, which is described as bionic honeycomb structure (BHS). The numerical simulation of the bionic model was carried out using nonlinear finite element solver Abaqus/Explicit. The energy absorbing properties of BHSs with various kinds of size were contrasted and discussed. In addition, a parameter study under axial loading with a different impact velocity was investigated.

## 2. Materials and Methods

### 2.1. Specimens Selection and Preparation

The beetle species of *Coccinella septempunctata* is adopted for the microstructure observations of the elytra. The species were collected from Beijing, China and then stored in laboratory (temperature = 20°, relative humidity = 50%). Just before performing the observation, all beetles were terminated at room temperature. Although the modifications of the wings may be occurred in the drying process, the high-quality images still could be obtained. After drying, the morphological investigations has been carried out by removing the elytra and hind-wing of each specimen.

### 2.2. Scanning Electron Microscopy

The specimens have been immersed in an alcoholic liquid for 30 h. Then the elytra and hind-wings of the beetles have been cut off by surgical knife. The preparations have been fixed onto SEM-holders (scanning electron microscope holders) and sputter-coated with gold-palladium (8 nm) and observed by a scanning electron microscope (EVO18, Zeiss, Germany) at 2.00 KV. The image measurements of microstructures were performed by using Digimizer Image Analysis Software version 5.3 (MedCalc, Ostend, Belgium).

### 2.3. Microstructures of Fiber Layers

Composite structures have excellent overall mechanical performance in terms of high strength, and impact and wear resistance. The mechanical properties of composites depend on the fiber content and the stacking method. Open literature review indicates that many beetle elytra have similar fiber layer structures, although the stacking methods are quite poorly understood, with authors only reporting that different fiber layers possess different undefined orientations. Most of the biological structures have an endocuticle showing the obvious presence of macro-fibers, in which the layers are stacked along the vertical direction. However, the approximate range of the angle of the layers could be estimated from the SEM images. In the present work, we identify for the first time the stacking angle, the arrangement mode, and the thickness of the layer structure of the *C. septempunctata* L. elytra.

**Figure 2:**
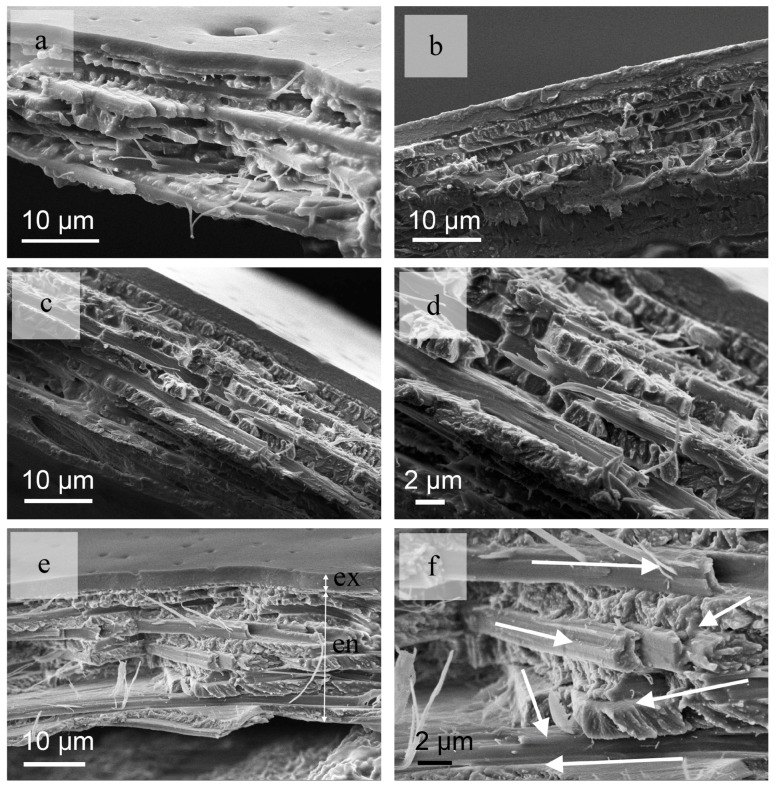
The cross-section view SEM (scanning electron microscopy) images of fiber layers from the *C. septempunctata* L. elytra. (**a**–**c**) show the full fiber layers; (**d**) shows higher magnification sections of fiber layers; (**e**) shows the thickness of the exocuticle (ex) and the endocuticle (en) in the *C. septempunctata* L. elytra; (**f**) shows higher magnification sections of endocuticle.

The thickness of the exocuticle (ex) and the endocuticle (en) of the *C. septempunctata* L. elytra are about 3 μm and 20 μm, respectively (Figure 2a–e). The thickness of each ply is essentially same at ~ 2 μm (Figure 2f). The layers are closely arranged and no gap is apparent between the fiber layers (Figure 2e,f). If we consider the basic direction of the fibers at 0° and 90°, the angles from the bottom to the top of the fiber layers appear to follow a stacking sequence similar to 0°/60°/−30°/30°/−60°/0°/90°/−30°/60°/−60° (Figure 2e). The angles of the bottom and the penultimate layers are 60°; if consider two layers as one single unit, the equivalent composite structure can be considered as made from five units. The angle between two adjacent units is 30°.

**Figure 3:**
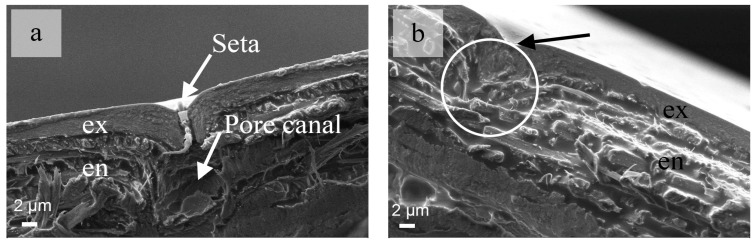
Cross-sectional SEM images of the *C. septempunctata* L. elytra. (**a**) seta and pole canal in the cuticle and (**b**) deformation of fiber layers (in white circle).

SEM images show that a large number of seta distributed on the surface of the elytra (Figure 3a,b). The distance between every two seta is about 30 μm (Figure 3a). The cuticle of the elytra have several pore canals penetrating through the exocuticle. There is a consistent one-to-one connection between each seta and pole canal. The pore canal breaks the continuity of the fiber layers in the endocuticle (Figure 3a). The break in the endocuticle also affects the mechanical properties of the fiber layers. In the various types of beetles, the size and density of the pore canals are different. In a previous study, the existence and contribution of the pole canals have been neglected and the whole fiber layer has been considered as a complete and solid structure. From an engineering application standpoint, it is interesting to understand how to reduce the influence of these breaks in the fiber layers on the mechanical characteristics of any bioinspired composite structure.

### 2.4. Microstructure of the Honeycombs

**Figure 4:**
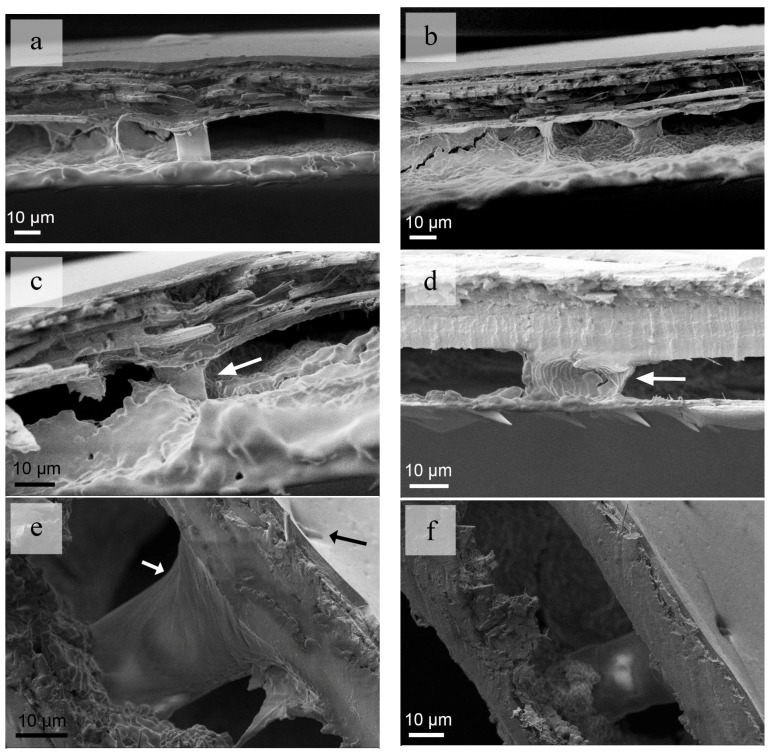
Honeycomb structures in the *C. septempunctata* L. elytra. (**a**,**b**) columns in the elytra; (**c**) column partially immedrsed in the foam; (**d**) internal structure of a column; (**e**) spiral surface of acolumn; (**f**) column and seta.

The honeycombs are presented in the epidermis structure (Figure 4a–c). According to previous work, the column connects the upper and lower layers to prevent their separation and make the forewings possess high inter-laminar strength. The columns also appear to possess a hollow structure (Figure 4d), and this is in accordance to previous observations in open literature. The hollow structure contributes to the reduction of the weight and makes the flight of the insect easier. At the end of a column, a spiral structure could be clearly observed (Figure 4e). Seta could also be detected above the column (Figure 4e,f), and the density of column could be speculated according to the position of seta or the pore canal.

## 3. Beetle-Based BHS

### 3.1. Structural Crashworthiness Criteria

Four indicators are adopted to indicate the energy absorbed properties of the bionic thin-walled structures [37]. The first one is *SEA* (specific energy absorption) which shows the energy absorption characteristics of a structure. *SEA* represents the ratio of *EA* (total absorbed energy by a structure) to the mass of structure:

(1) $$SEM=\frac{EA\;}{M}$$

The absorbed energy equals the area under force versus displacement curve:

(2) $$EA=\int_{0}^{d}F(x)\;dx$$

where *F* indicates the force under axial loading and *d* denotes the displacement under axial crushing. For an structure, the *CLE* (crash load efficiency) is defined as follow:

(3) $$CLE=\frac{MCF\;}{MIF}\times 100\%$$

where *MIF* means the maximum value of force in the curve under loading. The *MCF* (mean crush force) can be calculated as:

(4) $$MCF=\frac{EA\;}{d}$$

The large value of acceleration obtained in early stages of deformation will result in a significant increase of inertia force. Owing to the low wave propagation speed of the examined structure, a highly non-homogeneous state of deformation will be observed during impact loading. The condition which requires the inclusion of inertia stresses should be added into the analysis [38]. However, to simplify the model for better comparison of energy absorption characteristics of bionic structures, the discussion of inertia force in this model is neglected.

### 3.2. The Design of BHSs

**Figure 5:**
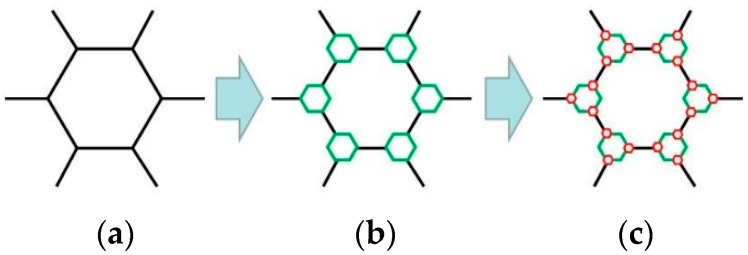
Unit cell of an evolved honeycomb: (**a**) the original cell; (**b**) the first order; and (**c**) the second order.

**Figure 6:**
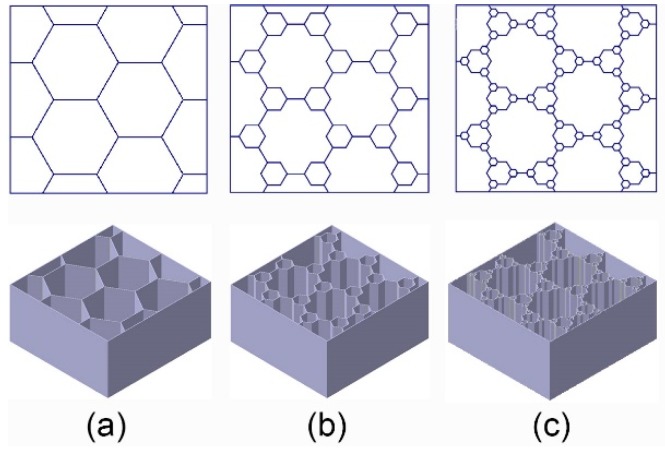
Three types of bionic structures with different filling methods of honeycombs: (**a**) BHS, original honeycombs; (**b**) BHS-1, filling mode with first-order; (**c**) BHS-2, filling mode with second-order.

Figure 5 shows three types of unit cells of evolved honeycomb. Considering the processing manufacture, the cell-wall thicknesses (*t_i_* = 1 mm) of each level of hierarchy remain the same. In this study, three kinds of BHSs with different filling methods inspired by beetle elytra internal structure were developed as shown in Figure 6. The BHS with original honeycombs is a conventional cellular structure. BHS-1 and BHS-2 are honeycombs with first order filling mode and second order filling mode, respectively. To investigate the energy absorption properties of the three kinds of hierarchy level, structures with equal areas of cross-section and equal length have been established. The geometric size of honeycombs strongly affect the mechanical behavior of the structure under crushing. In this work, the distance *d* which means the wall and the center of one hexagonal unit cell is chosen as the design variables. It ranges from 0 mm to 8 mm in BHS-1 with the interval of 2 mm and ranges from 0 mm to 3 mm in BHS-2 with the interval of 1 mm.

### 3.3. Mechanical Behavior of the BHS’s Material

**Figure 7:**
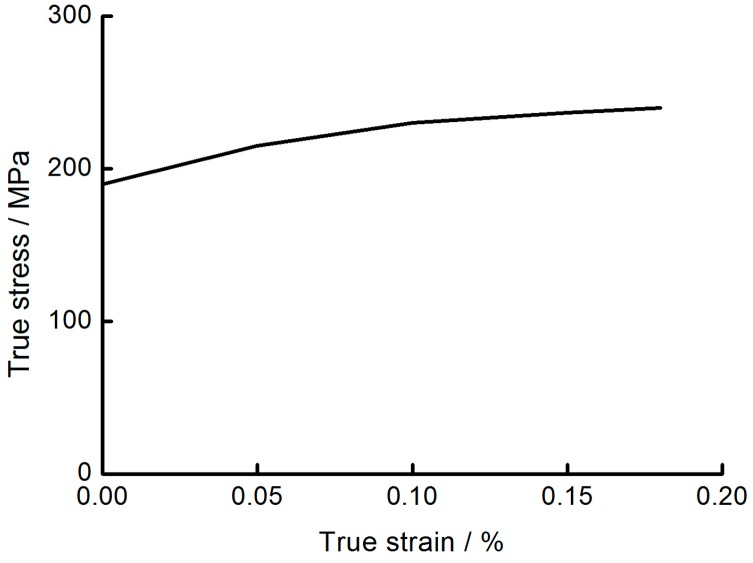
True stress versus true strain curve for AA6063.

The base material of all numerical examples has been adopted to aluminum alloy AA6063 T6 with the density of 2.7 × 10^3^ kg/m^3^, the initial yield stress of 162 MPa, a Young’s modulus of 67.9 GPa, the ultimate stress of 191 MPa, and Poisson’s ratio of 0.3 (Table 1) [39]. The models were established with MAT24 in Ls-Dyna (Livermore Software Technology Corp., Livermore, CA, USA). This above material shows strain rate insensitivity, and the true stress-strain value of AA6063 is showed in Figure 7 [40]. 

## 4. Numerical Simulations

### 4.1. Finite Element Modeling

**Figure 8:**
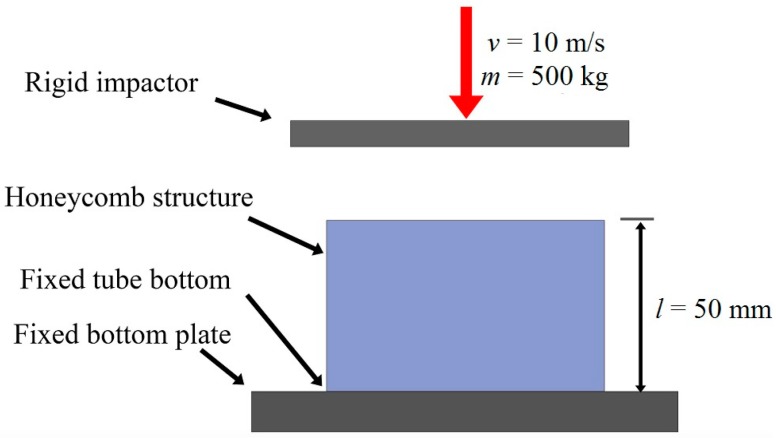
Schematic of the finite element model subjected to the axial loading.

The boundary conditions of BHS FE model is shown in Figure 8. The 4-node shell elements is adopted to divide the mesh in the case of large deformation analysis [41]. The impactor with initial velocity of 10 m/s and the weight of 500 kg is set as rigid body. The advancements in automotive safety requirements have indicated a need for improving performance of automotive crashworthiness. While the bionic thin-walled structures which studied in this manuscript is suitable for replacing the some energy absorbed components in the car. So the values of weight and speed of impactor are consisted with the vehicle impact requirements and they are also effectively provide safety for the passengers and drivers. The point-surface contact caused by the crushing and buckling of the structure’s wall under impact loading is considered in the numerical calculation [42]. The dynamic and static friction coefficients during crashing are set as 0.2 [43]. The reduced integration and the stiffness-based hourglass control are adopted to prevent volumetric locking and zero energy deformation mode happening [44].

**Figure 9:**
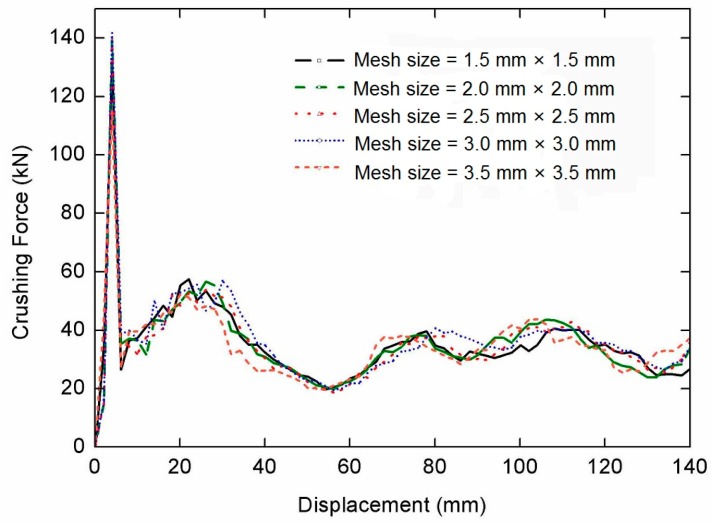
Crushing force versus displacement curves of FE models with five kinds of mesh sizes.

The commercial software CATIA V5R21 (Dassault Aviation, Paris, France) is adopted to create the finite element models. Hypermesh 12.0 (Altair Engineering Inc., Troy, NY, USA) is used to set meshing, loading conditions, material properties, and boundary conditions. An elastic-plastic material model with Von Mises isotropic plasticity algorithm is adopted to indicate the constitutive behavior of the thin shell element. The numerical simulations are calculated by the explicit nonlinear FE software Ls-Dyna (Livermore Software Technology Corp., Livermore, CA, USA). The curve graphs are refined by Origin Pro8 (OriginLab Corporation, Northampton, VA, USA) and the post-processing is performed by using Hypergraph 12.0 (Altair Engineering Inc., Troy, NY, USA). The curves for the crushing force versus displacement with five kinds of mesh elements size are shown in Figure 9. The difference between curves of and 2.0 mm × 2.0 mm is relatively small. To reduce the expense of calculation, the element size of 2.0 mm × 2.0 mm has been used for this study.

### 4.2. Validation of the FE Model

**Figure 10:**
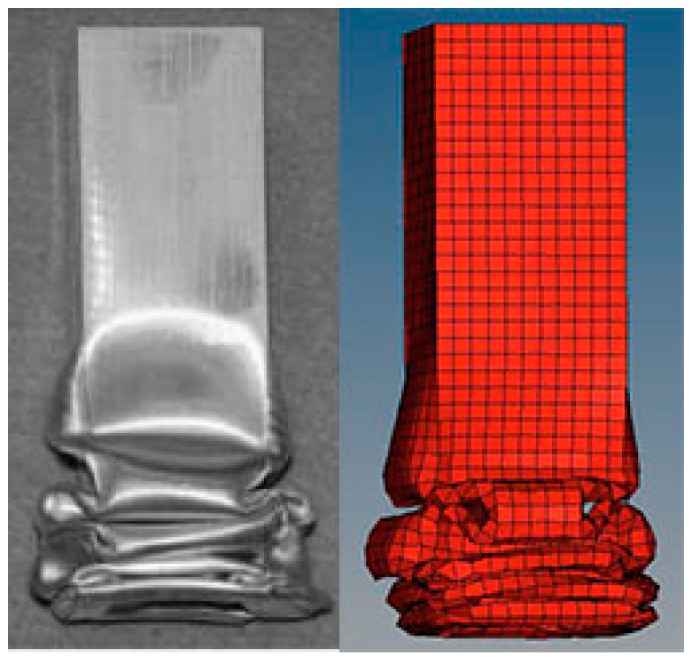
Comparison of deformation patterns of structure between experimental and numerical results.

**Figure 11:**
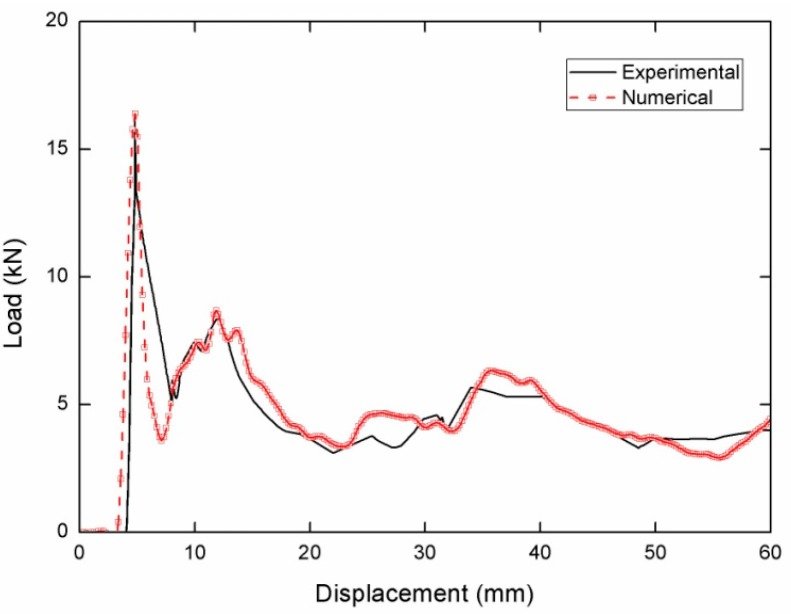
Comparison of force versus displacement curves between experimental and numerical results.

Lee et al. [45] have performed investigation on tubes under low velocity impact. The aluminum AA6063 tubes with 200 mm lengths has been used in their experiment. The impact velocity and weight of cross head were 7.02 m/s and 40 kg, respectively. These values are consisted with the vehicle impact requirements and they are also effectively provide safety for the passenger and drivers [46]. To validate the effect of numerical simulation, the same columns with the equal parameters are produced in this paper. The differences of deformation patterns and crushing curves between the simulations and the experiments are shown in Figure 10 and Figure 11. The crushing mode produced by FE simulations agrees well with the one from the experiments as shown in Figure 10. In addition, the curve of the force versus displacement strongly agrees with the one from the experiments as shown in Figure 11. The above results indicate our FE modelling approach could be used in investigating the characteristic of the BHSs in this study.

### 4.3. Comparison of Energy Absorption Properties of BHS-1 with Different Filling Cell Size

**Figure 12:**
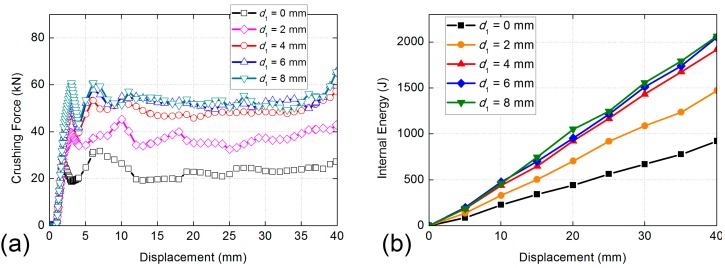
Comparisons of absorbed energy properties of BHS-1 with different filling honeycombs size: (**a**) crushing force versus displacement curves; (**b**) absorbed energy versus displacement curves.

The initial crushing forces of five cases of BHS-1 all obtain a peak value at the same time, then decrease obviously in different degrees, as shown in Figure 12a. Then the curves of all cases show small amplitude oscillations. The maximum value of BHS-1 is the case of *d*_1_ = 8 mm, while the mean crushing force of cases of *d*_1_ = 6 mm and *d*_1_ = 8 mm are very similar. The same trend of absorbed energy of different filling cell size is apparent, as shown in Figure 12b. The BHS without filling cell absorbed the minimum internal energy. With the increase of the filling cell size, the absorbed energy of BHS-1 is increased obviously. Although the internal energy is constantly growing, the trend of growth is slowing gradually. The internal energy of cases of *d*_1_ = 6 mm and *d*_2_ = 8 mm are both about 2000 J, and the different between them is not obvious. That means the case of *d*_1_ = 6 mm and *d*_2_ = 8 mm are very similar at the view of energy absorbing ability. Table 2 shows that the SEA of cell size of *d*_1_ = 6 mm is 66.79% higher than that of *d*_1_ = 0 mm and 15.48% higher than that of cell size of *d*_1_ = 2 mm, respectively. Compression force efficiency of cell size of *d*_1_ = 6 mm is 7.95% higher than that of cell size of *d*_1_ = 0 mm and 4.16% lower than that of cell size of *d*_1_ = 4 mm, respectively. In the view of SEA, the cell size of *d*_1_ = 6 mm is better than other cell size.

### 4.4. Comparison of Energy Aabsorption Characteristics of BHS-2 with Different Filling Cell Size

**Figure 13:**
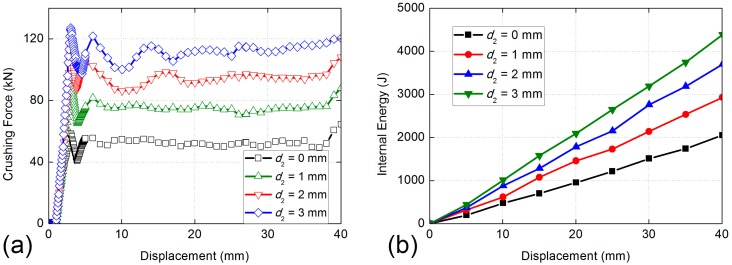
Comparisons of absorbed energy properties of BHS-2 with different filling honeycombs size: (**a**) crushing force versus displacement curves; (**b**) absorbed energy versus displacement curves.

The initial crushing forces of four cases of BHS-2 all get the peak value at the same time, then decrease rapidly with different degrees, as shown in Figure 13a. Then the curves of all cases go into slightly shock period. The maximum value of BHS-2 is the case of *d*_2_ = 3 mm, while the gaps of crushing force between all cases are relatively close. The same trend of absorbed energy of different filling cell size is obvious, as shown in Figure 13b. The BHS-1with *d*_1_ = 6 mm absorbed the minimum impact energy. With the increase of the filling cell size, the absorbed energy of BHS-2 is increased obviously. Unlike the trend of energy absorbing ability of BHS-1, the gaps of internal energy curves of different sizes of BHS-2 change closely with the increase of filling cell size. The internal energy of cases of *d*_2_ = 0 mm and *d*_2_ = 1 mm are 2000 J and 3000 J, respectively. The internal energy of cases of *d*_2_ = 2 mm and *d*_2_ = 3 mm are both more than 3500 J. Table 3 shows that the SEA of the cell size of *d*_2_ = 3 mm is 44.02% higher than that of *d*_2_ = 0 mm and 4.86% higher than that of cell size of *d*_2_ = 2 mm, respectively. Compression force efficiency of cell size of *d*_2_ = 3 mm is 11.72% higher than that of cell size of *d*_2_ = 0 mm and 5.79% higher than that of cell size of *d*_2_ = 2 mm, respectively. In the view of *SEA* and *CFE*, the cell size of *d*_2_ = 3 mm is better than the other cell sizes.

**Figure 14:**
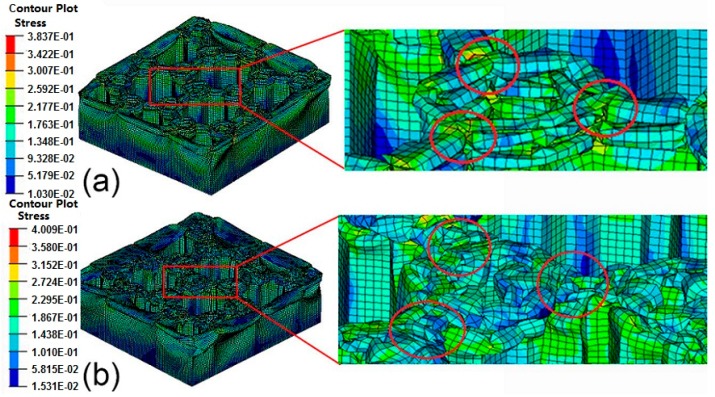
The deformation and stress contour of BHS under axial crushing: (**a**) BHS-1; (**b**) BHS-2.

The structural deformations and stress contours of BHS-1 with *d*_1_ = 6 mm and BHS-2 with *d*_2_ = 2 mm under low velocity loading are shown in Figure 14. The buckling of hollow thin-walled tubes occurs from the top to the bottom along the vertical direction in all numerical simulations. The red circle means the three connections of honeycomb corner in BHS-1, while it is observed that the stress of the connections is higher than that of other structures. In BHS-2, it is difficult to obtain that the stress changes of honeycomb corner. The structure is crushing and the wall of the corner is linked together.

### 4.5. Comparison of Energy Absorption Properties of BHSs with Different Impact Velocity

**Figure 15:**
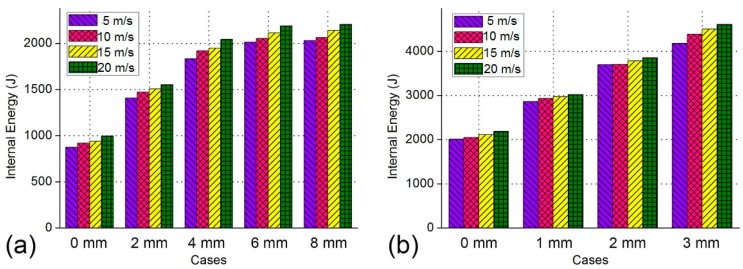
The absorbed energy of bionic honeycomb structures with different impact velocity: (**a**) BHS-1; (**b**) BHS-2.

To investigate the impact velocity of the BHS, a parametric study has been carried out by employing the impact velocity from 5 m/s to 20 m/s. Figure 15a,b show the energy absorption versus different velocity of BHS-1 and BHS-2, respectively. Figure 15a shows the energy absorption properties of BHS-1 become better gradually with the increase of the impact velocity. It is obvious that the impact velocity increases 5 m/s, the internal energy will increase by 2%–5%. Similarly to trend of crushing force curve, the energy absorption ability of cases of *d*_1_ = 6 mm and *d*_2_ = 8 mm are very close. Figure 15b shows the energy absorption ability of BHS-2 increase moderately with the growth of the impact speed. It could be obtained that the impact velocity increases 5 m/s, the internal energy will increase by 0.5%–4%. It can be also observed that the energy absorption property of case of *d*_2_ = 3 mm is the best of all cases.

## 5. Conclusions and Discussion

In this work, the biology layers of beetle elytra has been observed by scanning electron microscope, and the stacking methods of composite materials has been discussed. The staking sequence of biology layers has great significance for the design of composite laminates. The pole canal and honeycombs have also viewed by SEM. The finite element model has been established according to biologic structures in beetle elytra. The energy absorbing honeycomb structures with filling cellular cell of different hierarchy orders was built inspired by elytra internal structure. Then comparing the energy absorbed ability of BHS-1 and BHS-2 with the different filling cell size by employing nonlinear FE software. According to the numerical results, it could be obtained that the absorbed energy of BHS-1 is increased obviously with the increase of the filling cell size. The internal energy of cases of *d*_1_ = 6 mm and *d*_2_ = 8 mm are both about 2000 J, and the different between them is not obvious. The internal absorbing ability of cases of *d*_1_ = 6 mm and *d*_2_ = 8 mm are both better than other cases. Unlike the trend of energy absorbing ability of BHS-1, the gaps of internal energy curves of different sizes of BHS-2 change slightly with the increase of filling cell size. The absorbed energy of case of *d*_2_ = 3 mm which more than 3500 J has the best energy-absorbing property. Then the parameter study was carried out to study the influence of the impact velocity on energy absorption behaviors of BHS. The result shows that the impact velocity increases 5 m/s, the internal energy of BHS-1 and BHS-2 will increase by 2%–5% and 0.5%–4%, respectively.

The above research shows that the bionic structure inspired by a beetle elytra filled with a honeycomb structure of second hierarchy order mode has the best energy absorption properties under crushing. The honeycomb sandwich material investigated in this work could be adopted as a replaceable one in the vehicle design to improve energy absorption ability. Future works will focus on the manufacturability of this kind of bionic honeycomb material.

## Figures and Tables

**Table 1:** Mechanical behaviors of AA6063 for the bionic structures.

Density (kg/m^3^)	Young’s Modulus (GPa)	Yield Stress (MPa)	Ultimate Stress (MPa)	Poisson’s Ratio
2700	67.9	162	191	0.3

**Table 2:** Energy-absorbing behaviors of BHS-1 with different cell size.

Cell Size	*P_m_*/kN	*P_max_*/kN	*E_int_*/kJ	*m*/kg	*SEA* (kJ/kg)	*CFE*/%
0	23.263	31.714	0.919	0.074	12.421	73.353
2	36.860	45.620	1.471	0.082	17.939	80.801
4	48.519	58.723	1.919	0.091	21.088	82.624
6	52.237	65.969	2.051	0.099	20.717	79.185
8	52.852	66.545	2.063	0.108	19.101	79.423

**Table 3:** Energy absorbing behaviors of BHS-2 with different filling cell sizes.

Cell Size	*P_m_*/kN	*P_max_*/kN	*E_int_*/kJ	*m*/kg	*SEA* (kJ/kg)	*CFE*/%
0	52.2375	65.9690	2.051	0.099	20.717	79.185
1	75.0829	93.9902	2.930	0.112	26.161	79.884
2	95.3310	114.002	3.699	0.130	28.454	83.622
3	112.560	127.234	4.386	0.147	29.837	88.467
